# Investigating Thematic Roles through Implicit Learning: Evidence from Light Verb Constructions

**DOI:** 10.3389/fpsyg.2017.01089

**Published:** 2017-06-30

**Authors:** Eva Wittenberg, Manizeh Khan, Jesse Snedeker

**Affiliations:** ^1^Language Comprehension Lab, Linguistics Department, University of California, San DiegoSan Diego, CA, United States; ^2^Department of Psychology, Harvard UniversityCambridge, MA, United States

**Keywords:** thematic roles, light verb constructions, semantics, syntax, argument structure, eye tracking, syntactic alternations, implicit learning

## Abstract

The syntactic structure of a sentence is usually a strong predictor of its meaning: Each argument noun phrase (i.e., Subject and Object) should map onto exactly one thematic role (i.e., Agent and Patient, respectively). Some constructions, however, are exceptions to this pattern. This paper investigates how the syntactic structure of an utterance contributes to its construal, using ditransitive English light verb constructions, such as “Nils gave a hug to his brother,” as an example of such mismatches: Hugging is a two-role event, but the ditransitive syntactic structure suggests a three-role event. Data from an eye-tracking experiment and behavioral categorization data reveal that listeners learn to categorize sentences according to the number of thematic roles they convey, independent of their syntax. Light verb constructions, however, seem to form a category of their own, in which the syntactic structure leads listeners down an initial incorrect assignment of thematic roles, from which they only partly recover. These results suggest an automatic influence of syntactic argument structure on semantic interpretation and event construal, even in highly frequent constructions.

## Introduction

Thematic roles have been a foundational notion in linguistics for 50 years, ever since Gruber's (1965) seminal study on lexical relations. In psycholinguistics and language acquisition, the psychological reality of thematic roles has seen renewed interest in recent years, in particular through studies of unusual mappings between syntax and semantics, and how their acquisition and processing might shed light on the broader architecture of the language faculty (Chang et al., 2003; Bornkessel et al., 2006; Noble et al., 2011; Primus, 2012; Hartshorne et al., 2015; Rissman et al., 2015; and many others).

Among the processing studies of unusual mappings between syntax and semantics are those on light verb constructions (Piñango et al., 2006; Briem et al., 2009; Wittenberg and Piñango, 2011; Wittenberg et al., 2014). Consider Example (1):

(1) a. Nils gave a hug to his brother.
b. Nils hugged his brother.
c. Nils gave a book to his brother.

Example (1a) is a light verb construction that denotes the same event type as (1b), but uses the same surface syntax as (1c; Butt, 2010 and many others note that there is a wide variety of light verb constructions within and across languages, but we focus on the particular type in (1) for the purpose of this study). There is considerable debate about the representation of light verb constructions in linguistic theory, and about their thematic role structure that guides interpretation. The problem is that there is a non-homomorphism between the number of syntactic constituents (three: subject, and two objects) and event roles (two: *Nils, brother*) in light verb constructions such as (1a).

Traditionally, three broad families of approaches have been considered:

(1) Different Syntax (than a canonical ditransitive construction like “give a book”): This solution proposes that light verb constructions like (1a) have a fundamentally different syntactic representation than non-light constructions like (1c) (Hale and Keyser, 1993, 2002; Kearns, 1998; Gallmann, 1999; Jung, 2002; Folli et al., 2004). In these approaches, the light noun (*kiss* in *give a kiss*) forms part of the predicate and assigns thematic roles, such that (1a) and (1b) are semantically equivalent in their event type, each having two roles (Agent and Patient of *kiss*).

(2) Different Semantics (than a fully transparent transitive Base Verb, like “to kiss”): A second possibility is that light verb constructions are canonical transfer events, but the object of transfer (the Theme) is an action, and the whole event is understood metaphorically (Newman, 1996; Bruening, 2015). In this case, both the syntactic structure and the thematic roles of the light verb construction (1a) would be the same as that of non-light constructions (1c), with three noun phrases corresponding to the three thematic roles Source, Goal, and Theme.

(3) Different Mapping (than either Base Verb or Non-Light constructions): Other theorists (Jackendoff, 1974, 2002; Baker, 1989; Butt, 2010; Müller, 2016) have claimed that there is no syntactic difference between light and non-light constructions, but that the thematic roles in light verb constructions come from both the light verb and the light noun, in a phenomenon called “Argument Sharing.” According to this account, the verb *give* in non-light constructions such as in (1c) conveys the literal meaning of handing something over, but in light verb constructions like (1a), the same verb only signals a general sense of transfer, while the event nominal *kiss* contributes the event type itself (see Ramchand, 2014, for a conceptually similar account). Thus, Nils acts not only as the Agent of the verb *give*, but also as the Agent of the direct object *hug*, while *his brother* is both the Recipient of the verb and the Patient of the object.

According to this account, the event is constructed as having two roles, since the semantic structure of *kiss* calls for an Agent and a Patient; but the light verb *give* still exerts its influence by introducing a third role (*kiss* as a Theme of *give*). Thus, this proposal predicts that comprehenders entertain multiple event structures at once, resulting in a hybrid event construal unpredicted by either the Different Semantics or the Different Syntax account.

Wittenberg and Snedeker's (2014) found initial support for the Different Mapping hypothesis in a categorization experiment. In their study, participants sorted visual events (pictures on cards) and later linguistically described events according to the number of thematic roles. While canonical Agent-Patient and Source-Goal-Theme events were consistently sorted into the two- and three-role category, respectively, light verb constructions were split between these categories. This result is predicted for the different-mappings hypothesis, since under this hypothesis two sets of mappings are available for categorization and the observed split categorization may reflect this tension between candidate categories, but it is unexpected for current syntactic accounts of light verb constructions, and calls for further validation.

However, Wittenberg and Snedeker's (2014) conclusions were limited by the task they used. To teach participants to sort based on the number of roles, they relied on detailed instructions: They explicitly introduced the concept of thematic roles. Then, participants sorted pictures according to number of thematic roles, and received feedback on those training items. Thus, their findings could reflect participants' deliberate strategies and metacognitive intuitions about the experiment itself, rather than the more immediate, less explicit representations that underlie naturalistic language use.

Our goal here is to test the prediction that light verb constructions lead to unusual event construal, using an entirely implicit measure of categorization, in a task that does not require introducing the concept of thematic roles, and in a context where no feedback is provided. Such a finding would provide additional support for the Different Mappings hypothesis (and an empirical challenge for competing theories).

**Figure 1:**
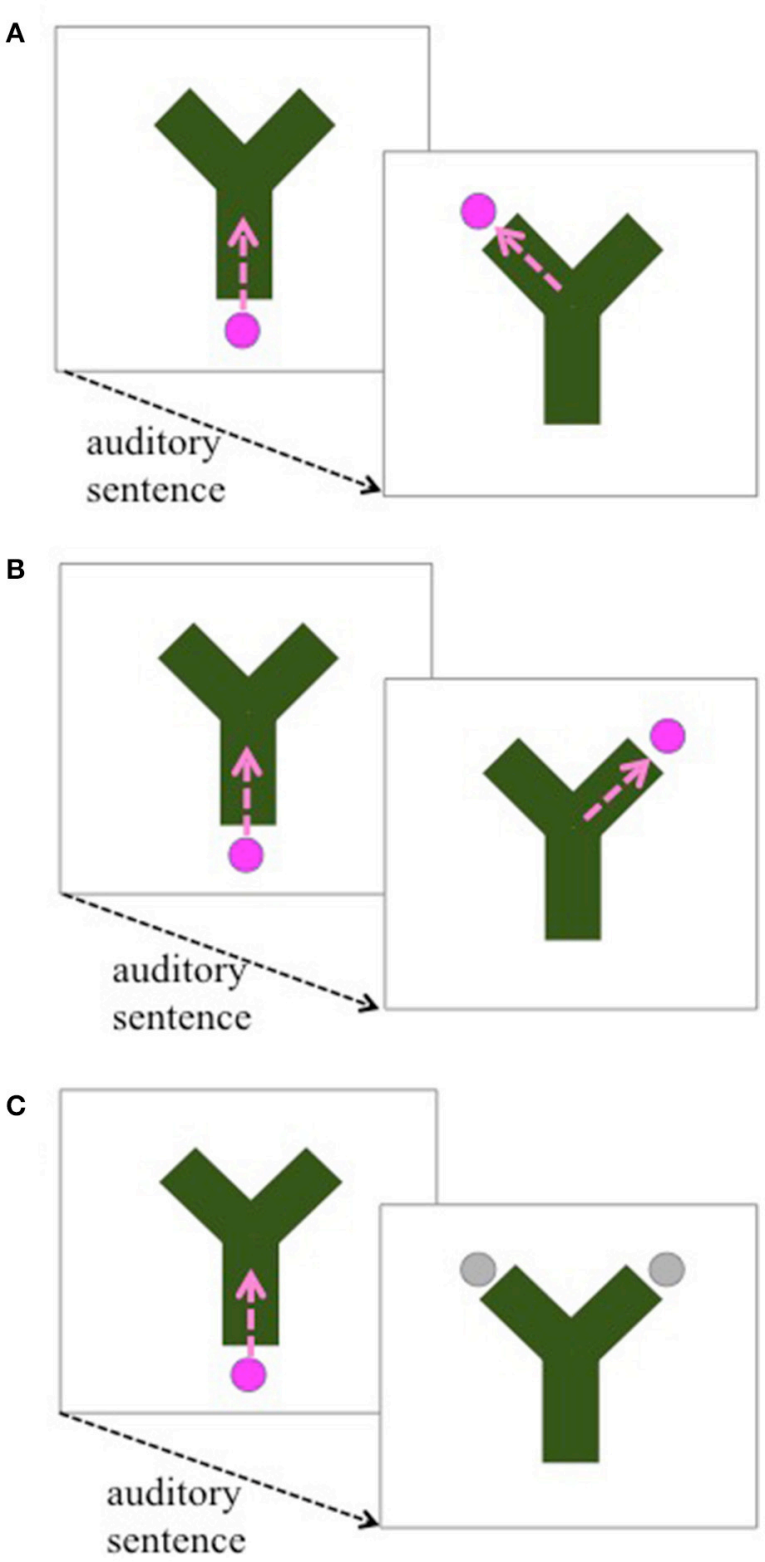
Start and end points of the videos accompanying each auditory stimulus sentence. In “click”-trials, Two- and Three Role sentences (75% of the trials), the ball entered the green tube (path shown by the dashed arrow), and either re-emerged on the left side, or the right side **(A,B**). There was a consistent match between side and number of roles (counterbalanced across participants). In “guess”-trials (25% of remaining trials, including base and light verb constructions), the ball did not re-emerge; instead, participants had to guess where the ball would have landed if it had re-emerged by clicking on one of the two possible landing sites **(C)**.

Our method is loosely based on Rohde and Horton's (2014) implicit categorization paradigm, which in turn was inspired by infant anticipatory looking studies (McMurray and Aslin, 2004). On each trial a Y-shaped tube was shown on the screen. A ball entered the tube at its base, just as an auditory sentence began. After the sentence ended, the ball reemerged on the top left or top right side of the Y, and participants' task was to click on it as quickly as possible (see Figure 1). Unbeknownst to the participants, one side was consistently associated with two-role events, while the other was consistently associated with three-role events (counterbalanced across participants, Figures 1A,B).

On critical trials, including all light verb construction trials, the ball “got stuck,” and participants had to guess the side where it would have emerged (Figure 1C). This was done so that participants would not get any feedback about how to categorize light verb constructions. Thus, we had two dependent measures of implicit categorization based on the number of thematic roles: anticipatory looks, and clicks to one side or the other.

Our predictions are simple:

1. If people can learn to categorize events based on thematic roles, they should click on and look more to the two-role side after hearing two-role sentences, and vice versa for three-role sentences.
2. If light verb constructions differ from non-light constructions in their syntax (different-syntax hypothesis) such that light nouns are part of the predicate, looks and clicks to light verb constructions should pattern with looks and clicks to two-role sentences. If light verb constructions are understood as metaphorical transfer events (different-semantics hypothesis), they should pattern with three-role sentences. If light verb constructions differ in their syntax-semantics mapping (different-mapping hypothesis), looks and clicks should be at chance, and different from both two- and three-role events, essentially replicating Wittenberg and Snedeker's (2014) results.

## Experiment

### Methods

#### Participants

Thirty two English speakers (Age: 18–25) from Harvard University participated in the study for course credit or monetary compensation. All participants learned American English as their native language, with five subjects learning an additional language before age 10. All subjects had normal or corrected to normal hearing and vision.

#### Materials

We used Wittenberg and Snedeker's (2014) set of 20 item pairs that consisted of light verbs (1a) and their base verb forms (1b)^1^. All light verb sentences used *give*; the event types that were described were, roughly, either events of touch (*kiss, hug, kick*), or communication (*call, answer, warning*). For each light verb construction, e.g., (1a, 2a), there was a corresponding base verb construction, e.g., (1b, 2b). In all light verb sentences, the verb was followed by the direct object and then by the prepositional object (cf. 2a). This was done such that the patient/recipient role was heard at the same time for both two-role, three-role, and light verb construction sentences.

(2) a. The robber gave a warning to his buddies.
b. The robber warned his buddies.

These pairs were matched on naturalness: On a scale from 1 (unnatural) to 9 (very natural), Wittenberg and Snedeker's (2014) mean naturalness ratings, obtained by 40 English native speakers, were 7.3 for Base Verb sentences, and 6.7 for Light Verb sentences [*F*_(1, 18)_ = 0.42. *p* > 0.52]. As training items, we used 228 sentences, half of which had two thematic roles (2a) while the others had three (2b). Those verbs were taken from Levin (1993) from verb classes that allowed no alternation between two and three arguments, and thus strongly lexically entailing two or three roles, respectively.

The training sentences varied on several dimensions in order to discourage counting of noun phrases, tracking syntactic structure, or making generalizations based on the concreteness or abstractness of the objects in a sentence. Thus, approximately half of the Two- and Three-Role items were declarative sentences, and half were questions (e.g., 3a and 3b). About half of them had a concrete Theme, and half of them had an abstract one (e.g., 3c). Finally, half of them described more entities than there were thematic roles (3c and 3d) to discourage an object counting strategy. The two-role sentences were designed to be the same length by using longer names, or occasionally adding genitive objects to the direct object (3e). Note how these added characters made two-role sentences more alike to three-role sentences, thus biasing against a strong distinction between the two sentence types. In addition, the aspectual types of two-role sentences were quite heterogeneous, while three-role sentences were overwhelmingly telic. Thus, on a number of dimensions, two-role sentences were more heterogeneous than three-role sentences, leading us to expect stronger learning for three-role sentences (for a full stimuli list, please refer to https://github.com/ewittenberg/Tubey).

(3) a.Grandma Kennison grew marijuana plants.
b. Did Christian wire the money to Anna?
c. The sound technician synthesized the voices.
d. The porcelain vase contained some dried flowers.
e. Henrietta scolded the neighbor's daughter.

On average, the sentence ended 1,404 ms after the offset of the verb. A female native speaker of American English recorded the sentences.

### Procedure

There were two types of trials: click-trials and guess-trials. We instructed the participants to listen to the sentences, and to click on the ball as soon as it reemerged from the tube; this was the task for the non-critical trials (“click-trials,” Figures 1A,B). On the critical trials the participants were told that the ball was stuck, and gray circles indicated the two possible landing sites (“guess-trials”; Figure 1C; also see Supplementary Material). Their task was to quickly click on the site where they presumed the ball would have come out. The first 60 sentences were always click-trials, in order to implicitly train participants on the association between number of roles and landing sites; after that, sentences were presented randomly. All critical trials, that is, the 10 light verb trials, and the 10 base verb trials, and an additional 28 sentences (50% two-role, 50% three-role) were guess-trials.

The task never explicitly told participants the pattern that determined where the balls landed, nor did it give any feedback for the guess-trials, in order to avoid influencing their categorization of the light verbs in subsequent light-verb trials. Thus, unlike in Wittenberg and Snedeker (2014), participants were not trained to provide correct classifications. In fact, they were not aware at all to the purpose of the experiment: A post-test questionnaire revealed that participants suspected the purpose of the experiment was entirely orthogonal to the question asked in this study, e.g., participants suspected the experiment to be investigating gender bias, or moral judgments.

The experimental stimuli were presented using ePrime. To record eye movements, we used a Tobii eyetracker that sampled at 60 Hz (i.e., recording participants' eyes every 16.6 ms).

### Data analysis of eye-tracking data

The goals of the analysis were: (a) to see whether participants learned the implicit connection between the number of thematic roles and the location of the emerging ball by looking at the correct side; and (b) to determine whether light verb constructions evoked more looks to the two-role or the three-role side.

To this end, we divided the screen into two halves and coded looks to the two-role side as 1, looks to the three-role side as 0, and track loss as missing data. Data were analyzed over a 3,000 ms time window, divided into time bins of 100 ms, starting at the end of the verb, since usually, the argument structure of an SVO sentence is not predictable before the verb is encountered. We excluded trials with more than 50% track loss in the critical time window. For the remaining trials, we calculated the mean proportion of looks to the two-role side in each time bin and performed a log-odds transformation on these proportions.

Our analyses focused on four comparisons: First, to verify the effectiveness of the manipulation, we compared the two- and three-role trials; then, to test the different theories of light verb constructions, we compared light and base verb trials, light and three-role trials, and light and two-role trials.

Since we had no *a priori* hypotheses about when the looking patterns would diverge, we used a non-parametric permutation test to correct for multiple comparisons (Maris and Oostenveld, 2007). Our analysis procedure allowed us to detect all contiguous clusters of statistically reliable effects, and test whether those clusters would be likely to occur by chance. For each 100 ms time bin, from 0 ms following verb offset to 2,999 ms, we conducted a mixed-effect regression analysis on the log-odds of looking to the two-role side, with condition as fixed effect (e.g., two-role vs. three-role), and random intercepts for subjects and items.

The procedure for a given contrast was as follows: First, we found clusters of temporally adjacent 100 ms bins where the *t-*value for each bin was larger than 1.6 (a quite conservative value that has been customarily used for eye-tracking analyses of this kind; see e.g., Hahn et al., 2015, and Maris and Oostenveld, 2007 for a discussion). For each cluster, we summed the test statistics for each bin to determine a cluster-level test statistic. Then, we permuted the data: Trial labels for condition were randomly shuffled within a subject. Then we repeated the cluster-finding procedure and summation of test statistics on the permuted data, and extracted the largest summed test statistic from any clusters that were identified. These were later used to create empirical distributions, against which the clusters from the original data could be compared.

This was done 1,000 times in order to create the empirical distributions. Finally, we compared the clusters from the original data and to the appropriate empirical distribution. The *p*-value for each cluster was calculated as the proportion of permuted clusters with larger cluster-level test statistics than the test statistic of the observed cluster.

One advantage of this procedure was that the specification of the test statistic was orthogonal to the process by which a cluster is determined to be significant. This meant that we could capture shallow, long-lasting effects by using a *t*-value of 1.6 without increasing the chances of a false-positive result.

## Results

A post-test questionnaire confirmed that no participant correctly deduced the purpose of the experiment or the principle behind the ball landing sites. Our analyses focused solely on the guess-trials, that is, the trials where the ball did not come out and participants had to guess the landing site.

### Mouse clicks

We analyzed mouse clicks with a logistic mixed-effects model, using Condition (two roles, three roles, base verb or light verb) as fixed effects, random slopes for subjects, and random intercepts for items.

**Figure 2:**
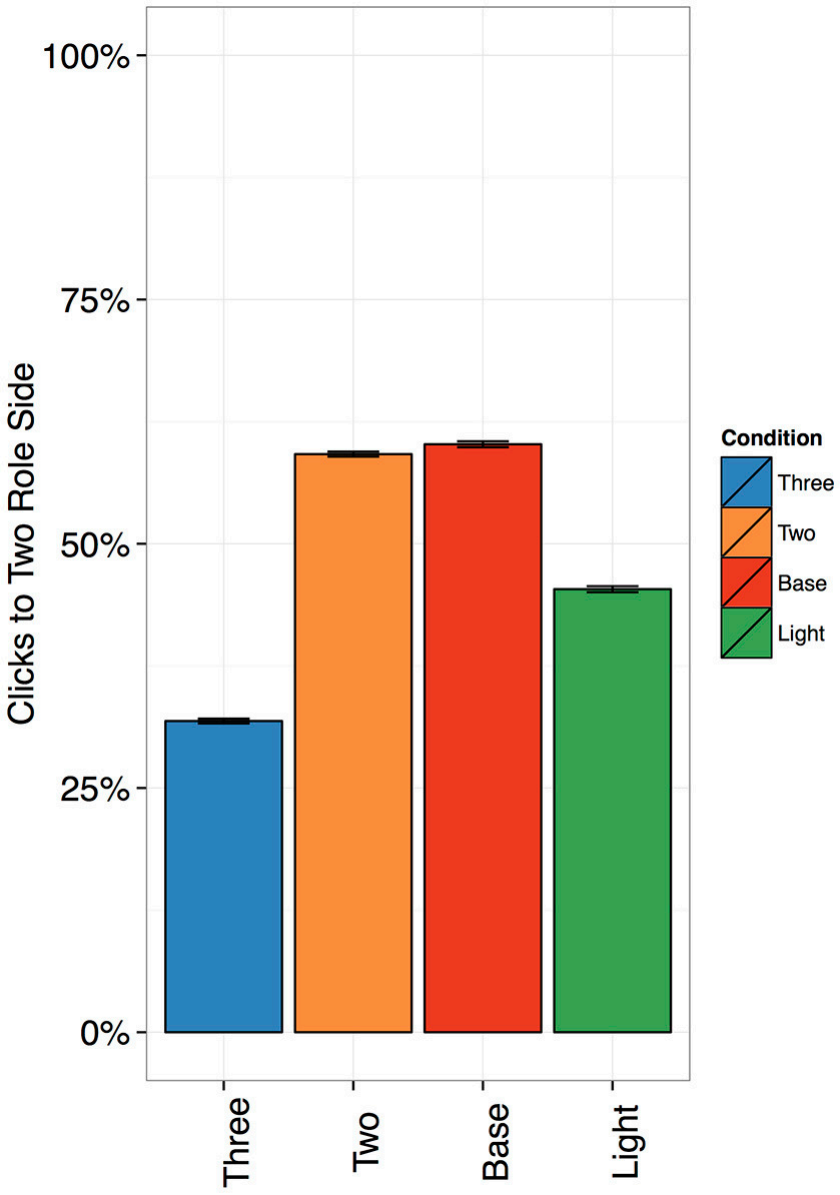
Percentage of clicks to the two-role side, with Standard Errors. The differences between two- and three-role sentences were significant, and so were the difference between light and two-role sentences and light and three-role sentences.

Participants implicitly learned to distinguish two-role and three-role sentences (Figure 2): They clicked on the two-role side 55% of the time for two-role sentences (SD: 20%), vs. 34% of the time for three-role sentences (SD: 19%). This difference was significant: β = 1.27, *z* = 139.53, *p* < 0.00001. For the base verb sentences, they clicked on the two-role side 57% of the time (SD: 22%), which was also significantly more than for three-role sentences (β = 1.41, *z* = 137.02, *p* < 0.00001). Thus, the mouse click data show that participants learned to distinguish events based on the number of thematic roles, even when they were not explicitly taught to do so.

In the case of light verb constructions, they clicked on the two-role side 48% of the time (SD: 23%)—which was significantly more than for three-role sentences (β = 0.59, *z* = 59.42, *p* < 0.00001), and significantly less than for two-role sentences (β = −1.66, *z* = −7.27, *p* < 0.00001). Thus, light verb constructions patterned neither with two- nor three-role sentences.

In by-subjects *post-hoc t*-tests (applying the Bonferroni correction for multiple comparisons), three-role sentences were significantly different from chance (*p* < 0.00001), but neither of the other conditions were (all *p*s > 0.06). This was not predicted by any of the theoretical accounts for the assignment of thematic roles in light verb constructions: We would have expected a clear difference from chance for both two- and three-role sentences. However, the lack of a significant difference for the two-role and base verb sentences may not be surprising, given that we had adapted them to match three-role sentences in length.

What is important to our analysis is that subjects learned to distinguish two- and three-role sentences *from each other*.

### Eye-movements

**Figure 3:**
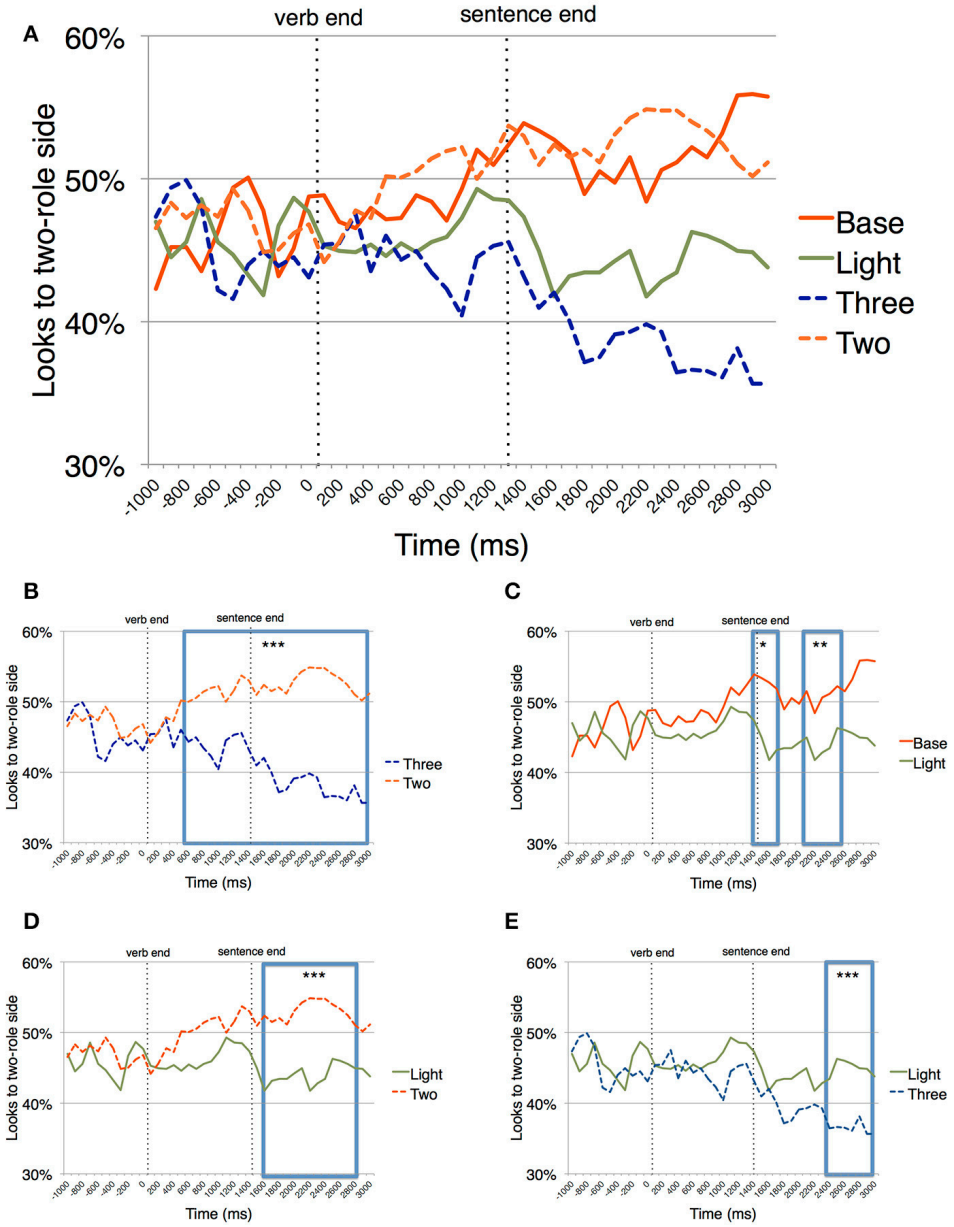
Percentage of looks to the two-role side over time. **(A)** While looks during the comprehension of two- and three-role sentences diverge early on, looks during the comprehension of light verb constructions do not pattern with either one. **(B)** Pairwise comparison between looks during comprehension of two- and three-role sentences. **(C)** Pairwise comparison between looks during comprehension of base and light sentences. **(D)** Pairwise comparison between looks during comprehension of two-role and light sentences. **(E)** Pairwise comparison between looks during comprehension of three-role and light sentences (Significance levels: ^***^p < 0.001; ^**^p < 0.01; ^*^p < 0.05).

Figure 3A shows the pattern of looks to the two-role side over time for all sentence types. Note that there was a general bias for looks toward the three-role side at the very beginning of the tracking period, suggesting that participants had a bias toward the three-role side. This is not surprising given the intentional heterogeneity of our two-role sentences; and just like in the mouse click data, the critical comparisons are between conditions, and not between any given condition and chance.

*Two- vs. Three-Role Trials* (Figure 3B). The permutation test identified a long cluster of significant differences between 600 and 2900 ms after verb offset (summed *t* statistic for cluster = 85.87, *p* < 0.0001), indicating that participants had implicitly learnt to associate each side of the screen with the correct landing site for the ball.

*Light vs. Base Trials* (Figure 3C). There was a marginally significant difference between the light and base verb trials in the time window between 2,100 and 2,500 ms after verb offset (summed *t* statistic for cluster = 9.64, *p* < 0.1), indicating that during most of the tracking time, the eye-movements for light verb constructions did not differ from eye-movements for base verb constructions. This is reassuring because those two types of sentences were conveying the same events: We can reasonably infer that the marginally significant difference late in the eye-tracking is due to confusion about the type of semantic roles, not because of syntactic differences between those two trial types.

*Light vs. Two-Role Trials* (Figure 3D). Between these two trial types, there was a long significant cluster of differences from 1,600 to 2,800 ms (summed *t* statistic for cluster = 30.43, *p* = 0.001). This shows that after the sentence ended, light verb constructions led to more looks to the three-role side than two-role trials did, again pointing to an uncertainty about the number of semantic roles.

*Light vs. Three-Role Trials* (Figure 3E). The permutation test identified a cluster of significant differences between 2,400 and 2,900 ms after verb offset (summed *t* statistic for cluster = 14.94, *p* < 0.04), indicating a late differentiation in eye-movements between light verb constructions and three-role trials.

In sum, what we find is that the ditransitive light verbs, but not the ditransitive three-role verb sentences, are alike two-role and base sentences for most of the time. This is unexpected if people simply categorize sentences based on syntactic argument structure, but expected if they categorize sentences based on number of semantic roles. On the other hand, light verb constructions were not different from three-role sentences either. That is unexpected if semantic structure alone guides interpretations.

## Discussion

This study validated Wittenberg and Snedeker's (2014) main finding, that people do not categorize events described by light verb constructions such as *giving a kiss* as either two role or three role events. Instead, light verb constructions are treated as intermediate between both options. Unlike Wittenberg and Snedeker's (2014) paradigm, our study did not rely on explicit instructions to categorize based on thematic roles. The task was implicit, participants were unaware of the regularity that governed the emergence of the ball, and thus the use of orthogonal strategies was unlikely.

Both our mouse click data and eye-tracking data show that light verb constructions exhibited a pattern different from both two- and three-role sentences: they seemed to be treated as being intermediate between both. This is particularly apparent in the eye tracking data, where at the end of the analysis window, the light verb trials showed a gaze pattern that was significantly different from both the two-role and three-role trials. The mouse click data showed that participants categorized light verb constructions significantly differently from both two- and three-role sentences, which is even stronger evidence for the intermediate status of light verbs than Wittenberg and Snedeker's (2014) data. All in all, our data, like Wittenberg and Snedeker's (2014) results, support the different-mapping hypothesis, which predicts that light verb constructions with *give* should pattern differently than two- or three-role events (Jackendoff, 2002; Wittenberg and Snedeker, 2014). In contrast, our results do not support the different-syntax or the different-semantics hypotheses, since these predict that light verbs should naturally group with either the two-role or three-role constructions, respectively.

In addition, the eye-tracking data are informative about the time-course of categorization: While two- and three-role sentences induced anticipatory looks that were clearly distinct before sentence end, light verb constructions only differ from any other category after the sentence ended. We take this an indication of uncertainty between two representations (two or three roles), before settling on a decision.

The different-syntax hypothesis is based on syntactic accounts of light verb constructions which assume a different underlying syntactic representation for light verb constructions and non-light constructions, such that light verbs and nouns jointly assign two thematic roles (Hale and Keyser, 1993, 2002; Gallmann, 1999; Jung, 2002; Folli et al., 2004). Thus, on these accounts, clicks and looks to the light verb constructions were predicted to pattern with the two-role sentences. The different-semantics hypothesis proposes that light verb constructions are non-light constructions, but with a metaphorical Theme (Newman, 1996). Thus, they were predicted to pattern with canonical three-role sentences. Finally, the different-mapping hypothesis assumed that light verb constructions straddle two overlapping sets of thematic roles, one from the light verb, and one from the light noun, resulting in an intermediate categorization pattern.

Of course, there is an important counterargument against this interpretation: Both the different-syntax and the different-semantics hypothesis could still be true, at the level of mental representation, but in the course of processing light verb constructions listeners could temporarily construct an incorrect analysis of the sentence, from which they never fully recover. On this account, light verb constructions cause semantic representations built from the surface structure that result in thematic misanalysis, and this initial misinterpretation lingers. Parallel phenomena have been noted by Ferreira and colleagues under the label of “good enough parsing” (Ferreira et al., 2001; Ferreira and Patson, 2007). For example, there is no ambiguity in the intended thematic structure of the syntactic garden-path sentence “While Anna dressed the baby spit up on the bed.” Anna is clearly dressing herself. Nevertheless, comprehenders will initially interpret the baby as the Theme, and misinterpretation will often linger affecting their later offline judgments.

A similar case could be made for our results: Perhaps, people first categorize light verb constructions as three-role events, because they share a *to*-PP, or because *give* commonly entails three syntactic arguments. This could be a likely contributor to our results: The light verb “give” activates its syntactic and semantic argument structure. The fact that people still categorize light verb constructions differently than both two- and three-role sentences merely points to the fact that comprehenders are sensitive to the aspects light verb constructions have in common with three-role sentences (syntactic structure, and one set of thematic roles) and those light verb constructions have in common with their two-role sentence counterparts (event structure and a second set of thematic roles). However, the *to*-PP did occur after the Theme (*kiss*, for light verb constructions); thus, people knew by the time they heard the *to*-PP that they were listening to a description of an Agent-Patient event.

Importantly, the different-mapping hypothesis is fully compatible with this scenario, since it recognizes that both the light verb and the light noun attempt to project their argument structures onto the event structure. Both the different-syntax and different-semantics accounts, however, would strain to explain such a mechanism: Light verb constructions are extremely frequent, unlike the garden-path sentences used in the shallow parsing literature, and adults have ample practice in using and comprehending these structures (Piñango et al., 2006; Wittenberg et al., 2014).

It is important to mention, however, that the results for the comparison items in this study (two- and three-role items) were not as clean as one would hope: While three-role sentences were reliably categorized correctly, the categorization for two-role sentences was at chance in the mouse-click data. As we discussed in the Methods section, this is likely due to the conservative design of our two-role sentence stimuli being as closely matched to three-role sentences in length and number of noun phrases as possible. In future studies using this methodology, one may circumvent this problem by using orthogonal strategies for increasing length in the shorter condition (in a case of categorizing thematic roles, adjectives or adverbs would be good candidates for lengthening, since they cannot bear thematic roles); or by increasing length variation in both conditions through a variety of syntactic devices.

This issue aside, we are hopeful that this case study and the methodology we used will be useful for further investigations of how the syntax-semantics interface is structured. One natural extension of this research program would be to repeat this study using light verb constructions with *do* (*do a dance*) or *take* (*take a shower*), which transform one-argument verbs into two-argument constructions. We predict similar effects for these constructions.

In addition, we have explored further effects of the syntactic frame onto the mental construal of events in light verb constructions: Wittenberg and Levy (2017) found that there are systematic changes in how people estimate event duration from base verbs to light verb constructions pairing telic light verbs like *give* with mass nouns (*give advice*), count nouns denoting durative events (*give a talk*), and count nouns denoting punctive events (*give a kiss*). Thus, investigating the effect of syntactic structure onto event construal seems to be a promising route.

## Ethics statement

This study was carried out in accordance with the recommendations of the Committee on the Use of Human Subjects (CUHS) at Harvard University, with written informed consent from all subjects. All subjects gave written informed consent in accordance with the Declaration of Helsinki. The protocol was approved by the Committee on the Use of Human Subjects (CUHS) at Harvard University.

## Author contributions

Conception and design of the work, Data analysis and interpretation, and Final approval of the version to be published: EW, JS, and MK. Data collection: EW and MK. Drafting the article: EW. Contributions to revisions of the article: JS and MK.

### Conflict of interest statement

MK is a current affiliate of Amazon, Inc., but the research was conducted before she joined the company. The other authors declare that the research was conducted in the absence of any commercial or financial relationships that could be construed as a potential conflict of interest.

## Footnotes

^1^Their stimuli are available under https://dataverse.harvard.edu/dataset.xhtml?persistentId=hdl:1902.1/19337, and our stimuli, together with frequency data and the Levin sources of verbs, are available under https://github.com/ewittenberg/Tubey.
